# Social Mobility and Mental Disorders at 30 Years of Age in Participants of the 1982 Cohort, Pelotas, Rio Grande Do Sul – RS

**DOI:** 10.1371/journal.pone.0136886

**Published:** 2015-10-08

**Authors:** Lenice de Castro Muniz de Quadros, Luciana de Avila Quevedo, Janaína Vieira dos Santos Motta, André Carraro, Felipe Garcia Ribeiro, Bernardo Lessa Horta, Denise Petrucci Gigante

**Affiliations:** 1 Graduate Program in Epidemiology, Federal University of Pelotas (Universidade Federal de Pelotas - UFPEL), Rio Grande do Sul, Brazil; 2 Graduate Program in Health and Behavior, Catholic University of Pelotas (Universidade Católica de Pelotas - UCPel), Rio Grande do Sul, Brazil; 3 Graduate Program in Organizations and Markets, Federal University of Pelotas (Universidade Federal de Pelotas - UFPEL), Rio Grande do Sul, Brazil; Medical University of Vienna, AUSTRIA

## Abstract

This study aimed to evaluate the relationship between mental disorders at 30 years of age and social mobility by formally testing three hypotheses: Risk Accumulation; Critical Period; and Social Mobility. The study was performed using data from the 30-year follow-up of the Pelotas Birth Cohort Study, conducted in 1982, and data from previous follow-ups. The tool used to evaluate mental health was the *Self Report Questionnaire* (SRQ-20). For the statistical analysis, the chi-square test with the Yates correction was used to estimate the prevalence of mental disorder, and the Poisson regression with robust variance was used to formally test the hypotheses according to the Risk Accumulation, Critical Period and Social Mobility Models. The analyses were stratified by gender. The prevalence of Common Mental Disorders (CMDs) was 24.3% (95% CI 22.9–25.7) when the whole sample was considered. The highest prevalence, 27.1% (95% CI 25.1–29.2), was found in women, and the difference between genders was significant (p < 0.001). CMDs were more frequent in participants who remained “poor” in the three follow-ups. In both men and women, the best fit was obtained with the Risk Accumulation Model, with p = 0.6348 and p = 0.2105, respectively. The results indicate the need to rethink public income maintenance policies. Finally, we suggest further studies to investigate the role of different public policies in decreasing the prevalence of mental disorders and thus contribute proposals of new policies that may contribute to the prevention of these disorders.

## Introduction

Problems related to mental health have a high prevalence in the general population. Mental illness is characterized in a complex way and involves social, cultural, politic and economic dimensions. This condition is expressed differently in social classes and in gender relations [1].

The expression of the social character of mental illness can be seen in its unequal distribution among men and women and among different social classes [1]. Health inequalities have been studied by researchers concerned with the epidemiology of mental illness [2,3]. When analyzing the relationship between social class and disease, most researchers conclude that the lower the social class, the higher the risk of psychiatric disorders [3–8]. In addition, longitudinal studies suggest an effect of economic status in early life on mental health in adulthood [3,9].

Among members from the 1982 cohort, a higher prevalence of common mental disorders (CMDs) was observed among individuals of the lowest income tertile at 23 years of age, regardless of socioeconomic status at birth, which suggests that mental health might be more strongly determined by the current socioeconomic status. However, exclusively for women, the same study showed that the family income at birth remained associated with CMDs even after adjustment for the current family income [10]. This cohort study comprises all children born in 1982 in the city of Pelotas whose mothers resided within the urban area of Pelotas municipality. Among all live-born children, less than 1% of the cases were lost, and the mother refused to participate in the study in less than 1%. Throughout the years, several follow-ups were performed with the following individuals from the cohort: in 1982, all children included in the perinatal study; in 1983, 1/3 of the cohort born between January and April; in 1984 and 1986, all children; in 1997, the residents of 27% of the city census sectors; in 2000, all males; in 2001, the same individuals as in 1997; from 2004 to 2005 and in 2000, all individuals from the cohort.

Concern about the association between socioeconomic status and health has been followed by discussion of the nature of the relationship between the two sets of concepts. For some researchers [11,12], the disease is the factor that may affect the socioeconomic status, whereas other authors [13] support reversing the causal relationship chain, suggesting that the low socioeconomic level leads to general health problems. The findings of Elovainio et al., 2011 [14], demonstrate that low socioeconomic levels in adults tend to define a trajectory of adverse change and cardiometabolic risk factors, especially adiposity, glucose metabolism, and onset of metabolic syndrome.

Longitudinal studies to analyze social differences and inequalities in health have been proposed to seek clarification about how exposure at different phases of life determines subsequent health statuses [15–19]. Social mobility is a strategy proposed to investigate and evaluate the socioeconomic trajectory and has been used to determine the relationship with health status and its risk factors in European countries [20–22].

To explain the effects of socioeconomic status throughout life on health outcomes, three hypotheses are proposed: Critical Period, Risk Accumulation and Social Mobility. The Critical Period hypothesis adopts the premise that the effect of exposure during a given period of life is the main determinant of risk and interferes with the outcome in different ways according to the period [23]. The Risk Accumulation hypothesis assumes that the gradual accumulation of exposure increases the risk of the outcome, and the Social Mobility hypothesis is based on the idea that changes in position between different categories of the social structure throughout life explain the outcome [23]. Using an approach that statistically compares the three models representing each of these hypotheses with a saturated model is the most adequate approach to the longitudinal analysis of lifelong exposure regarding a particular outcome [21,24].

Given the above, this study aimed to evaluate the relationship between mental disorder at 30 years of age and social mobility by formally testing the three hypotheses (Risk Accumulation, Critical Period and Social Mobility).

## Methods

This study used data from the 30-year follow-up of the 1982 Pelotas Birth Cohort study. The three maternity hospitals of the city were visited daily during the year of 1982, and the mothers of all 6,011 newborns who lived in the urban area were interviewed in the perinatal study. The 5,914 live births were examined and formed the original cohort. Subsequently, several follow-ups were performed, including subsamples or the whole cohort. The detailed methodology of these follow-ups is described in other published studies [25–28].

The 30-year follow-up that aimed to locate and interview all members of the 1982 cohort began in July 2012. The participants were asked to visit the Epidemiological Research Center for the data collection, which included questionnaires addressing demographic, socioeconomic, healthcare, physical activity, nutrition and mental health variables, in addition to physical examinations and collection of blood and serum samples.

The tool used to evaluate mental health was the *Self Report Questionnaire* (SRQ-20), which was designed by [29] and proposed by the World Health Organization to detect CMDs in the population. This instrument is a screening tool that consists of twenty questions with dichotomous yes or no answers. The evaluation is performed by investigating non-psychotic symptoms during the previous month, especially depression and anxiety. SRQ-20 consists of four questions about physical symptoms and 16 questions that address emotional issues. The translation and validation of the instrument for Portuguese were performed by [30], with a sensitivity of 83% and a specificity of 80%.

In this study, the presence of CMDs was defined using the number of positive answers to each of the SRQ-20 questions. Thus, women with eight or more positive answers in this scale were considered possible cases of minor psychiatric disorders. For men, the cut-off point was six or more positive answers. The prevalences of common mental health disorders are different between, genders and the SRQ 20 was validated in the Brazilian population with different cut-offs for men and women. The cut-offs suggested by Mari et al [31] were used in this paper.

Variables regarding family income were collected in all follow-ups. In the perinatal study, these variables were collected in five categories according to the minimum wage and were subsequently transformed into continuous variables using a process called allocation of income, which was based on characteristics of the family and the household, with principal component analysis (PCA) of four variables (public insurance system affiliation in delivery care, education, height and skin color of the mother of the cohort member). To analyze social mobility throughout life, in addition to the continuous variable obtained by PCA using data obtained in the perinatal study, information on family income collected in follow-ups that included the total sample of cohort members in 2004–5 (when participants of the cohort had a mean age of 23 years) and in 2012–13 (mean age of 30 years) was used. These data were collected continuously, in Brazilian Reais, and the distribution of all income variables in each of these three follow-ups was divided into tertiles, with the first classified as poor and the second and third as non-poor. Thus, the social mobility variable had eight defined categories: always poor, poor/non-poor/poor; poor/poor/non-poor; poor/non-poor/non-poor; non-poor/poor/poor; non-poor/non-poor/poor; non-poor/poor/non-poor, and never poor.

The eight categories constructed allowed description of the social mobility trajectory of members of the cohort at three points in their life cycle: at birth, at 23 years of age and at 30 years of age, i.e., *t*_*1*_, *t*_*2*_ and *t*_*3*_, respectively.

According to the methodology adopted by Mishra et al. [21], the social mobility trajectory was transformed into a dummy variable (*S*), which assumed the value “1” when the individual belonged to the second or third income tertile (non-poor category) and “0” when the individual belonged to the first income tertile (i.e., the poor category). Thus, *S* can be defined as the vector *S = (S*_*1*_..,*S*_*j*_ *)*, in which j = 1, 2 and 3 and the expected value of the interest variable, mental disorder (Y), can be expressed as a function of a linear combination of all *S*_*j*_, such that:

$$E(Y)=\alpha +\beta_{1}S_{1}+\beta_{2}S_{2}+\beta_{3}S_{3}+\theta_{12}S_{1}S_{2}+\theta_{23}S_{2}S_{3}+\theta_{13}S_{1}S_{3}+\theta_{123}S_{1}S_{2}S_{3}$$

The model described above considers that mental illness at 30 years of age is related to the income distribution in tertiles considering the three time points collected *(S*_*1*_,*S*_*2*_,*S*_*3*_ *)* and their interactions *(S*_*1*_ *S*_*2*_,*S*_*2*_ *S*_*3*_,*S*_*1*_ *S*_*3*_,*S*_*1*_ *S*_*2*_ *S*_*3*_ *)*, always compared with the expected value of Y (having a mental disorder) for the “always poor” trajectory. Considering this formulation as a starting point, hypotheses related to the Risk Accumulation Model, the Critical Period Model and the Mobility Model are tested.

In the Risk Accumulation Model, the greater the number of periods that a subject remains in the “poor” condition, the greater the risk of having a mental disorder. Thus, if all extreme cases are considered, a subject who remained “non-poor” for all three periods may have a different probability of having a mental disorder at 30 years of age than a subject who remained in the “always poor” condition for all three periods.

This hypothesis is represented by the following linear regression model:

$$E(Y)=\alpha +\beta \sum_{j=1}^{3}S_{j}$$

The above equation is obtained from substitutions into the previous equation treating the effect of *(S*_*1*_,*S*_*2*_,*S*_*3*_ *)* as identical regarding the mental disorder risk and assuming that socioeconomic fluctuations during life are not important. Thus, testing the Risk Accumulation Model hypothesis consists of performing a hypothesis test in which *H0*:*β*_*1*_ *= β*_*2*_ *= β*_*3*_ *;θ*_*12*_ *= θ*_*23*_ *= θ*_*13*_ *= θ*_*123*_ *= 0*.

The Critical Period Model considers that being “poor” during different phases of life may influence the outcome in different ways. This hypothesis considers the point in the life cycle at which the subject has the “poor” condition to be important. Thus, the current income is more important to the risk of having a mental disorder, regardless of the “poor” or “non-poor” condition at other points in life. In this model, different results obtained in the absence of the outcome due to the socioeconomic differences found among subjects only at moment *t*_*3*_ are compared. Thus, *Δ critical period = Y*_1**_ *—Y*_0**_. This model assumes that only the social status of the subject in adulthood is associated with the result of having a mental disorder or not, regardless of the trajectory. In this case, the linear regression model corresponds to the following equation:

$$E(Y)=\alpha +\beta_{3}S_{3}$$

In the Critical Period Model, the hypothesis test performed to verify its validity is *H*_*0*_:*β*_*1*_ *= β*_*2*_ *= θ*_*12*_ *= θ*_*23*_ *= θ*_*13*_ *= θ*_*123*_ *= 0*. Similarly, the same hypothesis can be tested for *t*_*1*_ and *t*_*2*_.

Finally, the Social Mobility Model compares the two adult time points of the subject to capture the effect of intergenerational mobility *(t*_*2*_ and *t*_*3*_ *)*. The hypothesis, in this case, is that the mental health condition of the subject can be influenced by socially ascending or descending. Therefore, in this model, social mobility involves both directions of change. Using the usual notations, the model is represented by the following linear regression equation:

$$E(Y)=\alpha +\beta_{2}S_{2}+\beta_{3}S_{3}+\theta_{23}S_{2}S_{3}$$

The hypothesis test to be performed is *H*_*0*_:*θ*_*23*_ *= -(β*_*2*_ *+ β3;β1 = θ12 = θ013 = θ123 = 0*. If the intergenerational mobility were considered, the model above would aggregate the change from moment *t*_*1*_ and could be rewritten as follows:

$$E(Y)=\alpha +\beta_{1}S_{1}+\beta_{2}S_{2}+\beta_{3}S_{3}+\theta_{12}S_{1}S_{2}+\theta_{23}S_{2}S_{3}$$

in which the interaction term *S*_*1*_ *S*_*2*_ captures the effect on the expected value of the intergenerational mobility Y. The hypothesis test to be performed here is *H*_*0*_:*β*_*2*_ *= (β*_*1*_ *+β*_*3*_ *);θ*_*12*_ *= θ*_*23*_ *= -β*_*2*_ *;θ*_*13*_ *= θ*_*123*_ *= 0*.

In the statistical analysis, the chi-square test with the Yates correction was used to estimate the prevalence of mental disorder, and the Poisson regression with robust variance3 was used to test the hypotheses according to the Risk Accumulation, Critical Period and Social Mobility Models. The analyses were stratified by gender because there is evidence in the literature that the risk of mental disorders is higher in female subjects [32]. The statistical analyses were performed using the software Stata 12.

The study was approved by the ethics committee of the School of Medicine of the Federal University of Pelotas (Universidade Federal de Pelotas—UFPEL), and an informed consent form was signed by all participants.

## Results

The first 30-year follow-up occurred between June 2012 and February 2013, and 3,701 members of the cohort were interviewed. Added to the 325 deceased participants of the cohort, this number reached a follow-up rate of 68% of the original cohort. Of the 3,701 members of the cohort, 1,914 (51.7%) were women, and approximately 66% lived with a partner. In total, 3,642 members of the cohort interviewed at 30 years of age answered the SRQ-20, including 1,757 (48.2%) men and 1,885 (51.8%) women.

Table 1 shows the prevalence of CMD in the total number of individuals and stratified by gender. This prevalence was 24.3% (95% CI 22.9–25.7) in the whole sample. A higher prevalence, 27.1% (95% CI 25.1–29.2), was found in women, and the difference between genders was significant (p < 0.001).

**Table 1:** Prevalence of CMD in men and women, Pelotas, 2012.

CMD	Total n = 3642	Men n = 1757	Women n = 1885
	% CI	%	%
**Yes**	24.3 (22.9–25.7)	21.3 (19.4–23.3)	27.1 (25.1–29.2)
**No**	75.7 (74.3–77.1)	78.7 (76.7–80.6)	72.9 (70.8–74.9)

Table 2 shows the eight possible income trajectories. The income was considered a dichotomous variable in which 0 represents “poor” and 1 “non-poor” individuals. The income distribution was described for the total sample and separately for men and women. The group with the best income, i.e., “non-poor”, included approximately 40% of participants of the study at the three time points studied. Among men, the lowest proportion was found in individuals who suffered a descending social mobility when compared to the 1982 and 2004 follow-ups and who remained in the lowest income group in 2012. Among women, the lowest proportion was found in individuals who remained in the “poor” group in the first two periods and who had ascending mobility in 2012.

**Table 2:** Distribution of the social mobility variable trajectory, Pelotas, 1982/2004/2012.

Year of follow-up			Total n (%)	Men n (%)	Women n (%)
1982	2004	2012			
0	0	0	320 (10.0)	126 (8.1)	194 (11.9)
0	1	0	199 (6.2)	93 (6.0)	106 (6.5)
0	0	1	183 (5.7)	102 (6.5)	81 (5.0)
0	1	1	299 (9.4)	171 (11.0)	128 (7.8)
1	0	0	244 (7.6)	83 (5.3)	161 (9.9)
1	1	0	335 (10.5)	165 (10.6)	170 (10.4)
1	0	1	288 (9.0)	148 (9.5)	140 (8.6)
1	1	1	1328 (41.6)	673 (43.1)	655 (40.1)

0 = Poor 1 = Non-Poor

Table 3 shows the presence of CMD at 30 years of age for each of the eight possible categories. Significant associations were found for men and women. CMD was more frequent in subjects who remained “poor” in the three follow-ups, and the lowest proportion of mental disorders was found in subjects who remained “non-poor” in all follow-ups.

**Table 3:** Prevalence of CMD according to Social Mobility, Pelotas, 1982/2004/2012.

Year of follow-up			Total n = 3191	Men n = 1558	Women n = 1633
1982	2004	2012	%	%	%
			P = < 0.001	P = 0.001	P = < 0.001
0	0	0	40.4	30.9	46.6
0	1	0	29.1	20.4	36.8
0	0	1	30.0	27.4	33.3
0	1	1	18.7	18.1	19.5
1	0	0	36.5	31.3	39.1
1	1	0	27.0	24.5	29.4
1	0	1	25.0	23.6	26.4
1	1	1	16.5	17.0	16.1

0 = Poor 1 = Non-Poor

Table 4 shows the analysis performed to test each of the three hypotheses (Risk Accumulation, Critical Period and Social Mobility) regarding mental disorder proposed in this study. For both men and women, the Risk Accumulation was the best-fit model, with p = 0.6348 and p = 0.2105, respectively. For men, the largest proportions of CMD are in subjects classified as “poor” in *t3*, regardless of the classification in previous periods, as indicated in the Critical Period Model. This result is not evident only in the condition in which “non-poor” was observed only in *t3*. Regarding women, the Risk Accumulation Model is clear, as all four situations (0 0 0, 0 1 0, 0 0 1 and 1 0 0) have the highest prevalence of mental disorder.

**Table 4:** Results of saturated and restricted tests according to the different hypotheses tested, Pelotas 1982/2004/2012.

Hypothesis	Statistic*	P-value
**Men**		
No effect	24.52	0.0009
Accumulation	4.31	0.6348
Critical period		
t1	21.16	0.0017
t2	11.81	0.0663
t3	9.36	0.1542
Intergenerational mobility	24.05	0.0002
Any mobility	21.47	0.0007
**Women**		
No effect	117.05	<0.001
Accumulation	8.40	0.2105
Critical period		
t1	77.13	<0.001
t2	74.74	<0.001
t3	31.60	<0.001
Intergenerational mobility	111.29	<0.001
Any mobility	113.41	<0.001

* Chi-square test

## Discussion

The prevalence rates of mental disorder found in this study and the difference found between men and women are consistent with results previously found in the same municipality with a slightly younger population [10,33]. It is worth noting that these rates were already present for members of this cohort at a mean age of 23 years [10], and although the effect of change in income from birth to 23 years of age had been presented, this study describes three different models for the longitudinal analysis of the effect of income on CMD. In addition, it is important to emphasize the relevance of evaluating the prevalence rates of mental disorder in different age groups, especially during early adult life, a time of decision making and personal and professional changes that may cause suffering and symptoms related to CMD. In a systematic review [34] performed between 1997 and 2009 on the general prevalence of mental disorder in the adult Brazilian population, these rates ranged from 20 to 56%. Relatively high prevalence rates were observed, and the use of more sensitive or more specific cut-off points in some studies that contribute to explaining the differences in these prevalence rates cannot be discarded.

Worldwide, problems related to mental health have been widely reported, indicating high prevalence rates among the general population [35]. Millions of people suffer from some type of mental disease, and this number is progressively increasing, especially in developing countries [36,37]. Mental disorders lead to individual suffering and have important socioeconomic implications because they may cause lost work days and burden health services36. The Pan American Health Organization (PAHO) and the World Health Organization (WHO) note that the prevalence of mental disorders in the population is increasing34 and estimate that approximately 450 million people around the world suffer from mental or neurobiological disorders, which represent the fourth leading cause of disability in the world population. From this perspective, this study evaluated mental disorders at 30 years of age by testing the association with the socioeconomic trajectory throughout the life cycle. For this purpose, the three following general hypothesis models were used: Risk Accumulation, Critical Period and Social Mobility, which aim to examine the longitudinal effects of economic status on any outcome.

Although there are different ways to evaluate social mobility [38,39], in general, studies analyze the influence of social mobility on health by occupational classification [40,41]. In this study, we used family income because occupational classification in Brazil does not have a clear and definitive definition and also because this information was not collected in the perinatal study (*t1*), which prevents construction of the social mobility trajectory based on occupational classification. Because this work is a longitudinal study, it has the advantage of not being influenced by time, preventing the memory bias that could occur, especially with questions regarding family income at birth in 1982.

This study is important for public health because data from follow-ups performed in developing countries at three time points with the same population and the follow-up rate found in this study are rare.

The SRQ-20 is a screening procedure for common mental health diseases, and it represents a limitation for the present study considering that neither psychotic symptoms nor substance abuse, among other mental diseases, could be identified by this instrument.

Instead of using a saturated analysis model [21], the hypotheses of risk accumulation, critical period and social mobility are suggested for studying the effects of socioeconomic trajectory on health outcomes [23,42–44].

Thus, this study presents the homogeneous effects of income and its mobility regarding gender because the best-fit model was the Risk Accumulation Model, for both men and women. However, when only the Critical Period Model was evaluated, *t3* had a reasonable fit (p = 0.1542) only for men. Of the three hypotheses tested, the results found here indicate that the Risk Accumulation Model is the best-fit model for men and women. Thus, the association between socioeconomic level and mental disorder occurred according to the number of periods in which the cohort member was in a situation of economic vulnerability, i.e., this association occurred when the cohort member remained in the “poor” category for longer periods of time. These results suggest that long-term policies regarding socioeconomic status could be more effective in decreasing the prevalence of mental disorder.

The results found in this study indicate the need to rethink public policies for income maintenance. Programs aimed at direct income transfer are common in Brazil, and social security systems based on retirement and unemployment insurance have been developed. However, these public policies focus on the effect on short-term income. Long-term economic measures should also be planned in Brazil and in other countries in South America. In Brazil, the Family Grant Program (Programa Bolsa Família) stands out as an advance in direct income transfer programs for the poor [45]. Finally, we suggest further studies to investigate the role of different public policies in decreasing the prevalence of mental disorder and thus contribute proposals of new policies that may contribute to the prevention of these disorders.

## References

[1] Ludemir AB. Desigualdades de classe e gênero e saúde mental nas cidades. [class and gender inequalities and mental health in cities] PhysisRevista de Saúde Coletiva. 2008;18: 451–467.

[2] Macintyre S. The patterning of health by social position in contemporary Britain: directions for sociological research. Soc Sci Med. 1986;23: 393–415. 3529428 10.1016/0277-9536(86)90082-1

[3] Melchior M, Moffitt TE, Milne BJ, Poulton R, Caspi A. Why do children from socioeconomically disadvantaged families suffer from poor health when they reach adulthood? A life-course study. Am J Epidemiol. 2007;166: 966–974. 17641151 10.1093/aje/kwm155 PMC2491970

[4] Dohrenwend BP. Socioeconomic status (SES) and psychiatric disorders. Are the issues still compelling? Soc Psychiatry Psychiatr Epidemiol. 1990;25: 41–47. 2406949 10.1007/BF00789069

[5] Lima MS, Soares BGO, Mari JJ. Saúde e doença mental em Pelotas, RS: dados de um estudo populacional. [mental health in Pelotas, Southern Brazil: data from a population-based cross-sectional survey] Rev Psi Clin. 1999;26: 225–35.

[6] Maragno L, Goldbaum M, Gianini RJ, Novaes HM, César CL. Prevalência de transtornos mentais comuns em populações atendidas pelo Programa Saúde da Família (QUALIS) no Município de São Paulo, Brasil. [prevalence of common mental disorders in a population covered by the Family Health Program (QUALIS) in São Paulo, Brazil]. Cad Saúde Pública. 2006;22: 1639–1648. 16832535 10.1590/s0102-311x2006000800012

[7] Marín-León L, Oliveira HB, Barros MB, Dalgalarrondo P, Botega NJ. Social inequality and common mental disorders. Rev Bras Psiquiatr. 2007;29: 250–253. 10.1590/S1516-44462006005000060 17891254

[8] Lima MC, Menezes PR, Carandina L, Cesar CL, Barros MB, Goldbaum M. Transtornos mentais comuns e uso de psicofármacos: impacto das condições socioeconômicas. [common mental disorders and use of psychoactive drugs: the impact of socioeconomic conditions]. Rev Saúde Pública. 2008;42: 717–723. 18604365 10.1590/s0034-89102008005000034

[9] Gilman SE, Kawachi I, Fitzmaurice GM, Buka SL. Socioeconomic status in childhood and the lifetime risk of major depression. Int J Epidemiol. 2002;31: 359–367. 10.1093/ije/31.2.359 11980797

[10] Anselmi L, Barros FC, Minten GC, Gigante DP, Horta BL, Victora CG. Prevalência e determinantes precoces dos transtornos mentais comuns na coorte de nascimentos de 1982, Pelotas, RS. [prevalence and early determinants of common mental disorders in the 1982 birth cohort, Pelotas, Southern Brazil]. Rev Saúde Pública. 2008;42 (Suppl2): 26–33. 10.1590/s0034-89102008000900005 PMC2667238 19142342

[11] Illsley R. Occupational class, selection and the production of inequalities in health. Q J Soc Aff. 1986;2: 151–165.

[12] West P. Rethinking the health selection explanation for health inequalities. Soc Sci Med. 1991;32: 373–384. 2024152 10.1016/0277-9536(91)90338-d

[13] Power C, Manor O, Fox J. Health and class: the early years. London: Chapman and Hall; 1991

[14] Elovainio M, Ferrie JE, Singh-Manoux A, Shipley M, Batty GD, Head J, et al Socioeconomic differences in Cardiometabolic factors: Social causation or health-related selection? Evidence from the Whitehall II Cohort Study, 1991–2004. Am J Epidemiol. 2011;174: 779–789. 10.1093/aje/kwr149 21813793 PMC3176829

[15] Tress W, Schwen-Harant T. Psychogenic illness and social mobility between generations. Psychother Psychosom Med Psychol. 1991;41: 1–5. 2017544

[16] Timms D. Gender, Social mobility and psychiatric diagnoses. Soc Sci Med. 1998;46: 1235–1247. 9572613 10.1016/s0277-9536(97)10052-1

[17] World Health Organization. The implications for training of embracing: a life course approach to health. Geneva: WHO; 2000

[18] Poulton R, Caspi A, Milne BJ, Thomson WM, Taylor A, Sears MR, Moffitt TE. Association between children’s experience of socioeconomic disadvantage and adult health: a life-course study. Lancet. 2002;360: 1640–1645 [10.1016/S0140-6736(02)11602-3] 12457787 PMC3752775

[19] Tiffin PA, Pearce MS, Parker L. Social mobility over the lifecourse and self reported mental health at age 50: Prospective cohort study. J Epidemiol Comm Health. 2005;59: 870–872. 10.1136/jech.2005.035246 PMC1732913 16166361

[20] Melchior M, Berkman LF, Kawachi I, Krieger N, Zins M, Bonenfant S, Goldberg M. Lifelong socioeconomic trajectory and premature mortality (35–65 years) in France: findings from the GAZEL Cohort Study J Epidemiol Community Health. 2006;60: 937–944 [10.1136/jech.2005.042440] 17053282 PMC1991279

[21] Mishra G, Nitsch D, Black S, De Stavola B, Kuh D, Hardy R. A structured approach to modelling the effects of binary exposure variables over the life course. Int J Epidemiol. 2009;38: 528–537. 10.1093/ije/dyn229 19028777 PMC2663717

[22] Tiikkaja S, Sandin S, Malki N, Modin B, Sparén P, Hultman CM. Social class, Social mobility and risk of psychiatric disorder—A population-based Longitudinal Study. PLOS ONE. 2013;8: e77975 10.1371/journal.pone.0077975 24260104 PMC3829839

[23] Hallqvist J, Lynch J, Bartley M, Lang T, Blane D. Can we disentangle life course processes of accumulation, critical period and social mobility? An analysis of disadvantaged socio-economic positions and myocardial infarction in the Stockholm Heart Epidemiology Program. Soc Sci Med. 2004;58: 1555–1562. 14759698 10.1016/S0277-9536(03)00344-7

[24] Motta JVS. Mobilidade social e fatores modificáveis para doenças crônicas não transmissíveis, um estudo longitudinal, Pelotas, RS. [Social mobility and modifiable factors for chronic non-communicable diseases in a longitudinal study, Pelotas, RS.]/ Thesis by Janaína Vieira dos Santos Motta; Adviser: Denise Petrucci Gigante.–Pelotas: UFPel. 2014;128 f.: il.

[25] Victora CG, Barros FC, Martines JC, Béria JU, Vaughan JP. Estudo longitudinal das crianças nascidas em 1982 em Pelotas, RS, Brasil: metodologia e resultados preliminares. [Longitudinal study of children born in Pelotas, Rio Grande do Sul, Brazil, in 1982. Metodology and preliminary results] Rev Saúde Pública. 1985;19: 58–68. 10.1590/s0034-89101985000100007 4081609

[26] Victora CG, Barros FC. Cohort profile: the 1982 Pelotas (Brazil) Birth Cohort Study. Int J Epidemiol. 2006;35: 237–242. 16373375 10.1093/ije/dyi290

[27] Barros AJD, Santos IS, Matijasevich A, Araújo CL, Gigante DP, Menezes AMB, et al Methods used in the 1982, 1993, and 2004 birth cohort studies from Pelotas, Rio Grande do Sul State, Brazil, and a description of the socioeconomic conditions of participants' families. Cad Saúde Pública. 2008;24 (Suppl 3): s371–s380. 18797712 10.1590/s0102-311x2008001500002

[28] Barros FC, Victora CG, Horta BL, Gigante DP. Metodologia do estudo da coorte de nascimentos de 1982 a 2004–5, Pelotas, RS. [methodology of the Pelotas birth cohort study from 1982 to 2004–5, Southern Brazil]. Rev Saúde Pública. 2008;42 (Suppl 2): 7–15. 10.1590/s0034-89102008000900003 PMC2671798 19142340

[29] Harding TW, de Arango MV, Baltazar J, Climent CE, Ibrahim HH, Ladrido-Ignacio L, et al Mental disorders in primary health care: a study of their frequency and diagnosis in four developing countries. Psychol Med. 1980;10: 231–241. 7384326 10.1017/s0033291700043993

[30] Mari JJ, Williams P. A comparison of the validity of two psychiatric screening questionnaires (GHQ-12 and SRQ-20) in Brazil, using Relative Operating Characteristic (ROC) analysis. Psychol Med. 1985;15: 651–659. 4048323 10.1017/s0033291700031500

[31] Mari JJ, Williams P. Misclassification by psychiatric screening questionnaires. J Chron Dis 1986;39: 371–378. 3700578 10.1016/0021-9681(86)90123-2

[32] Silva MT, Galvao TF, Martins SS, Pereira MG. Prevalence of depression morbidity among Brazilian adults: a systematic review and meta-analysis. Rev Bras Psiquiatr. 2014;36: 262–270. 25119639 10.1590/1516-4446-2013-1294

[33] Jansen K, Mondin TC, da Costa Ores L, de Mattos Souza LD, Konradt CE, Pinheiro RT, et al Transtornos mentais comuns e qualidade de vida em jovens: uma amostra populacional de Pelotas, Rio Grande do Sul, Brasil [common mental disorders and quality of life in youth: a population-based sample from Pelotas, Rio Grande do Sul, Brazil]. Cad. Saúde Pública [online]. 2011;27: 440–448. ISSN 0102-311X. 10.1590/s0102-311x2011000300005 21519695

[34] Santos ÉGd, Marluce S. Prevalência dos transtornos mentais na população adulta Brasileira: uma revisão sistemática de 1997 a 2009. [prevalence of mental disorders in the Brazilian adult population: a systematic review from 1997 to 2009]. J Bras Psiquiatr. 2010;59: 238–246.

[35] OMS. Organização Mundial de Saúde. Relatório sobre a saúde no mundo. Saúde mental: nova concepção, nova esperança. In: The world health report 2001—mental health: New understanding, new hope. Geneva; 2001.

[36] Menezes PR, de P. Epidemiologia Psiquiátrica. [principles of psychiatric epidemiology] In: Almeida OP, Laranjeira R, Dratcu L, editors Manual de Psiquiatria. Rio de Janeiro: GUANABARA-KOOGAN; 1995 pp. 43–54.

[37] Lopez AD, Murray CC. The global burden of disease, 1990–2020. Nat Med. 1998;4: 1241–1243. 9809543 10.1038/3218

[38] Pero V. Mobilidade social no Rio de Janeiro. [Social mobility in Rio de Janeiro]. Rev Econ Mackenzie. 2006;4: 136–153.

[39] Pero V, Szerman D. Mobilidade intergeracional de renda no Brasil. [intergenerational income mobility in Brazil]. Pesqui Planejamento Econ. 2008;38: 1–36.

[40] Ribet C, Zins M, Gueguen A, Bingham A, Goldberg M, Ducimetière P, Lang T. Occupational mobility and risk factors in working men: selection, causality or both? Results from the GAZEL study J Epidemiol Community Health. 2003;57: 901–906 [10.1136/jech.57.11.901] 14600118 PMC1732330

[41] Tehranifar P, Liao Y, Ferris JS, Terry MB. Life course socioeconomic conditions, passive tobacco exposures and cigarette smoking in a multiethnic birth cohort of U.S. women. Cancer Causes Control. 2009;20: 867–876. 10.1007/s10552-009-9307-1 19238563 PMC3704144

[42] Lawlor DA, Davey Smith G, Patel R, Ebrahim S. Life-course socioeconomic position, area deprivation, and coronary heart disease: findings from the British women's Heart and Health Study. Am J Public Health. 2005;95: 91–97. 15623866 10.2105/AJPH.2003.035592 PMC1449858

[43] Naess Ø, Claussen B, Thelle DS, Davey Smith G. Cumulative deprivation and cause specific mortality. A census based study of life course influences over three decades. J Epidemiol Comm Health. 2004;58: 599–603. 10.1136/jech.2003.010207 PMC1732810 15194723

[44] Singh-Manoux A, Ferrie JE, Chandola T, Marmot M. Socioeconomic trajectories across the life course and health outcomes in midlife: evidence for the accumulation hypothesis? Int J Epidemiol. 2004;33: 1072–1079. 15256527 10.1093/ije/dyh224

[45] Tavares P, Pazello E, Fernandes R, Camelo R. Uma avaliação do Programa Bolsa Família: focalização e impacto na distribuição de renda e pobreza. [an evaluation of the Family Grant Program (Programa Bolsa Família): targeting and impact on income distribution and poverty]. Pesqui Planejamento Econ. 2009;39: 25–58.

